# Multi–Omics Analysis of Key microRNA–mRNA Metabolic Regulatory Networks in Skeletal Muscle of Obese Rabbits

**DOI:** 10.3390/ijms22084204

**Published:** 2021-04-19

**Authors:** Yanhong Li, Jie Wang, Mauricio A. Elzo, Mingchuan Gan, Tao Tang, Jiahao Shao, Tianfu Lai, Yuan Ma, Xianbo Jia, Songjia Lai

**Affiliations:** 1 College of Animal Science and Technology, Sichuan Agricultural University, Chengdu 611130, China; lyh81236718@stu.sicau.edu.cn (Y.L.); wjie68@sicau.edu.cn (J.W.); ganmingchuan@stu.sicau.edu.cn (M.G.); ttly0524@gmail.com (T.T.); shaojiahao@stu.sicau.edu.cn (J.S.); ltf11064@gmail.com (T.L.); mayuan@stu.sicau.edu.cn (Y.M.); jaxb369@sicau.edu.cn (X.J.); 2 Department of Animal Sciences, University of Florida, Gainesville, FL 32611, USA; maelzo@ufl.edu

**Keywords:** microRNA, mRNA, metabolism, network, rabbit, skeletal muscle

## Abstract

microRNAs (miRNAs), small non-coding RNA with a length of about 22 nucleotides, are involved in the energy metabolism of skeletal muscle cells. However, their molecular mechanism of metabolism in rabbit skeletal muscle is still unclear. In this study, 16 rabbits, 8 in the control group (CON–G) and 8 in the experimental group (HFD–G), were chosen to construct an obese model induced by a high–fat diet fed from 35 to 70 days of age. Subsequently, 54 differentially expressed miRNAs, 248 differentially expressed mRNAs, and 108 differentially expressed proteins related to the metabolism of skeletal muscle were detected and analyzed with three sequencing techniques (small RNA sequencing, transcriptome sequencing, and tandem mass tab (TMT) protein technology). It was found that 12 miRNAs and 12 core genes (e.g., *CRYL1*, *VDAC3* and *APIP*) were significantly different in skeletal muscle from rabbits in the two groups. The network analysis showed that seven miRNA-mRNA pairs were involved in metabolism. Importantly, two miRNAs (miR-92a-3p and miR-30a/c/d-5p) regulated three transcription factors (MYBL2, STAT1 and IKZF1) that may be essential for lipid metabolism. These results enhance our understanding of molecular mechanisms associated with rabbit skeletal muscle metabolism and provide a basis for future studies in the metabolic diseases of human obesity.

## 1. Introduction

The metabolism and development of skeletal muscle are crucial in the process of animal growth. Additionally, the metabolism of energy for normal life activities is affected by a variety of growth, differentiation, and nutritional environment factors. An increasing number of people have suffered from obesity-related metabolic diseases in recent decades due to excessive intake of high–fat diets. Obesity also caused intramuscular metabolic disorders, such as mitochondrial disease, systemic inflammation, abnormal adipocytokine signal transduction, and excessive lipid accumulation [1,2,3]. Previous studies have shown that the metabolic regulation model of skeletal muscle is of great significance to the regulation of obesity [4]. In particular, the content of intramuscular fat in rabbits is relatively lower than in other livestock, indicating that it has unique patterns of muscle growth and metabolism. However, there have been few studies addressing the regulatory mechanisms involved in rabbit muscle growth and metabolism.

microRNAs (miRNAs) are a type of evolutionarily conserved short non-coding RNA with a length of about 20 to 23 nucleotides. They can bind to specific sites in the 3′-untranslated region (3′-UTR) of messenger RNAs (mRNAs) to regulate gene transcription. Numerous studies have shown that miRNAs are widely involved in the regulation of growth and development in skeletal muscle. For example, miR-27, miR-133a/b, miR-499a/b, miR-208a/b, and other miRNAs participate in the regulation of muscle differentiation and are primarily expressed in muscle tissues [5,6].

Skeletal muscle is closely associated with meat yield and other important economic characteristics in domestic animals. Multiple miRNAs and candidate genes active during various growth and development stages of muscle tissue have been identified through sequencing technologies. Studies have shown that a large number of miRNAs and genes are involved in growth and metabolism regulation during skeletal muscle development in pigs, cattle, sheep and poultry [7,8,9,10]. Further, the targeted regulatory relationship between miRNAs and mRNA plays a vital role in the process of mRNA transcription and protein translation [11]. miR-130b and miR-696 target the peroxisome proliferator-activated receptor-γ coactivator-1α (*PGC-1α*) gene to regulate skeletal muscle metabolism [12,13]. miR-499 targets the *PRDM16* gene to regulate adipogenic differentiation between muscle and adipose tissue [14]. miR-143 and miR-378 target the *IGFBP5* and *POLA2* genes, respectively, to regulate skeletal muscle satellite cell proliferation and differentiation [15,16]. However, miRNA–mRNA network regulation and genetic mechanisms of skeletal muscle growth and metabolism in rabbits are far from clear.

Thus, the objectives of this study were as follows: (1) to differentially screen expressed miRNAs and genes associated with rabbit skeletal muscle growth and metabolism in an obese group fed a high–fat diet and a control group fed a commercial diet; (2) to perform a regulatory network and functional analysis of miRNA–mRNA to help elucidate rabbit skeletal muscle growth and metabolic regulatory mechanisms.

## 2. Results

### 2.1. Phenotypic Difference and Small RNA Deep Sequencing Data from CON–G and HFD–G

Anatomical phenotypic differences (Supplementary Figure S1) showed that rabbits fed a high–fat diet contained large amounts of fat under the skin and on the viscera, which was consistent with previous studies, indicating that the high–fat diet achieved the expected obesity effect [17]. Small RNA from skeletal muscles of rabbits in CON–G and HFD–G were sequenced with a HiSeq 2500 (SE50) sequencer. A range of 10097828 to 10354071 clean reads of skeletal muscle samples from six rabbits (three from each group) was obtained. Low-quality and meaningless reads were filtered out; these reads had quality scores (QS) ≤ 5, and accounted for more than 50% of the complete reads, reads with 5′ joint contamination, reads with greater than 10% of unidentifiable base information, reads without a 3′ connector sequence and insertion fragment, and reads with mostly continuous missequenced polyA/T/G/C. 

**Figure 1:**
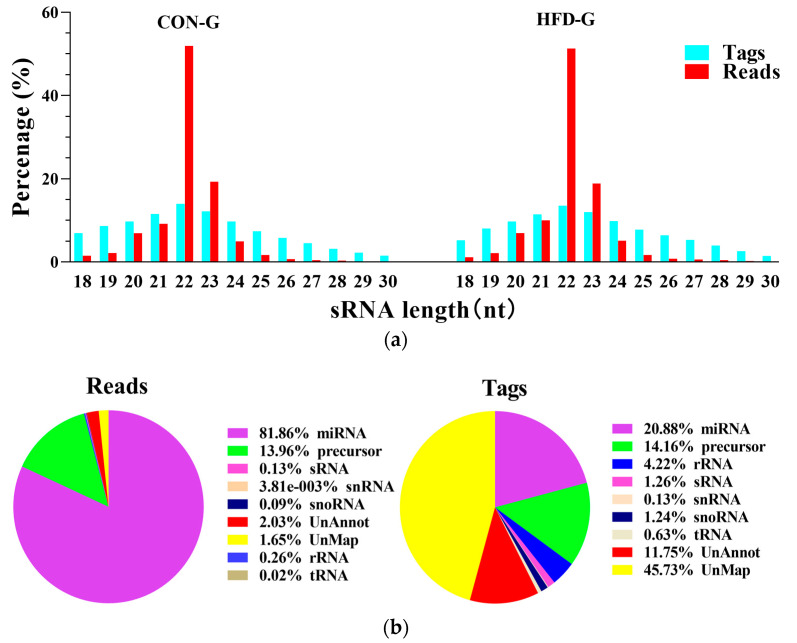
Quality analysis of small RNA sequencing. (**a**) Distribution and abundance of small RNA sequence lengths (18 to 32 nt) in clean reads of skeletal muscle tissue samples from rabbits in CON–G and HFD–G; (**b**) statistical distribution of small RNA annotations. Left, classification statistics of all small RNA reads; right, classification statistics of small RNA tags.

The data quality of small RNA sequences (18 to 32 nt) in the six skeletal tissue samples from CON–G and HFD–G is shown in Table 1. The number and length distribution of small RNA tags are presented in Figure 1a. Most of the small RNA sequence lengths were mainly concentrated in the range of 21 to 23 nt; 22 nt sequences were the most frequent, followed by 21 and 23 nt sequences. Subsequently, the unique small RNA reads were mapped to chromosomes by blasting the rabbit genome. Results showed that over 90% of the reads and 60% of the tags could be perfectly mapped to the rabbit genome. Finally, the type and number of sRNAs were classified into seven groups by using Bowtie2 software and the Rfam database (https://rfam.xfam.org/, accessed on 20 July 2020) to blast total sRNA tags. sRNAs are different from small RNAs, which are the total RNA species in the extracted RNA of muscle tissues. sRNAs are small, non-coding RNA species that regulate most cellular processes. In total, 81% of small RNA reads were identified as miRNAs and 13.96% as precursor RNA, 1.65% were unmatched, and the remaining ones were identified as ribosomal RNA (rRNA), small noncoding RNA (sRNA), small nuclear RNA (snRNA), small nucleolar RNA (snoRNA), or transfer RNA (tRNA) (Figure 1b). These results indicated the miRNA data were reliable for study.

### 2.2. Identification and Screening of Differentially Expressed miRNAs

**Figure 2:**
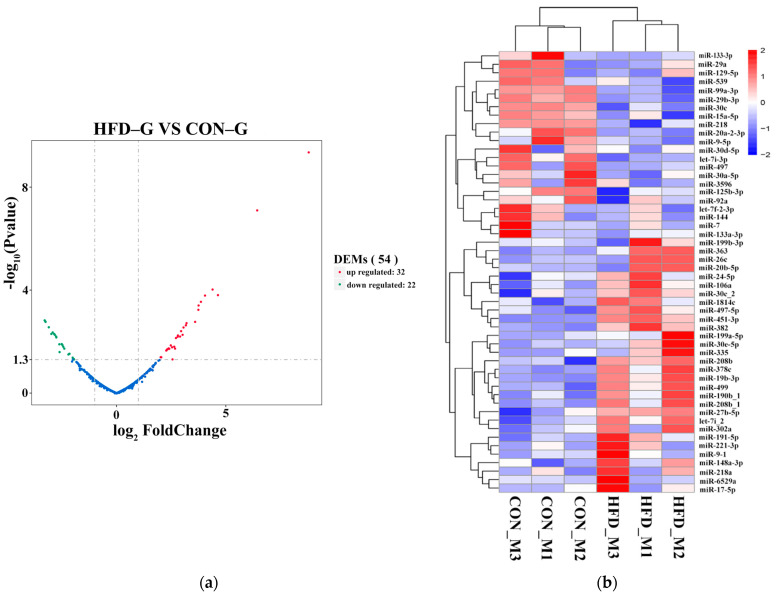
Map of identified DEMs in skeletal muscle samples from CON–G and HFD–G rabbits. (**a**) Volcano map of DEMs in muscle. Green, blue, and red represent significantly reduced miRNAs, non-significant miRNAs, and upregulated miRNAs, respectively. (**b**) Heatmap of DEMs. Blue and red represent downregulated and upregulated miRNA expression, respectively. Cluster analysis was conducted for sample and differential miRNAs.

A total of 1207 miRNAs (687 known mature miRNAs and 520 novel miRNAs) were identified in skeletal muscle samples from the CON–G and HFD–G rabbits. The known miRNAs were regarded as the main content and chosen for subsequent differential expression analysis because of the relative lower expression level of novel miRNAs. The differentially expressed miRNAs (DEMs) were the main target of this research to help understand changes in the metabolism of skeletal muscle in rabbits fed a high–fat diet. The DEMs from the CON–G and HFD–G rabbits were screened out using an EdgeR analysis with a |log2Foldchange (FC)| ≥ 1 and false discovery rate (FDR) < 0.05 as screening criteria (Figure 2a). The details of different expressed novel miRNAs are presented in Supplementary Table S1. In all, 54 DEMs (32 upregulated and 22 downregulated) were identified with the heatMap package for miRNA expression pattern cluster analysis (Figure 2b). These reflected the huge difference in miRNAs in rabbit muscle caused by obesity.

Subsequently, we chose the top 20 DEMs, 10 upregulated (miRNAs of miR-499-5p, miR-30e-5p, miR-363-3p, let-7i-3p, miR-19b-3p, miR-26c, miR-6529a, miR-148a-3p, miR-30c-5p, and miR-92a) and 10 downregulated (miR-30a-5p, miR-30d-5p, miR-125b-3p, miR-7, miR-99a-3p, miR-3596, let-7f-2-3p, miR-218, miR-20a-2-3p, and miR-133-3p) for further analysis (Table 2). Seven miRNAs, four upregulated (miR-499-5p, miR-30e-5p, let-7i-3p, and miR-26c) and three downregulated (miR-99a-3p, miR-3596, and miR-133-3p) had significant differential expression in the two rabbit groups. Twenty important DEMs in muscle, induced by obesity, were considered for further study.

### 2.3. Target Gene Prediction, Function Enrichment Analysis

**Figure 3:**
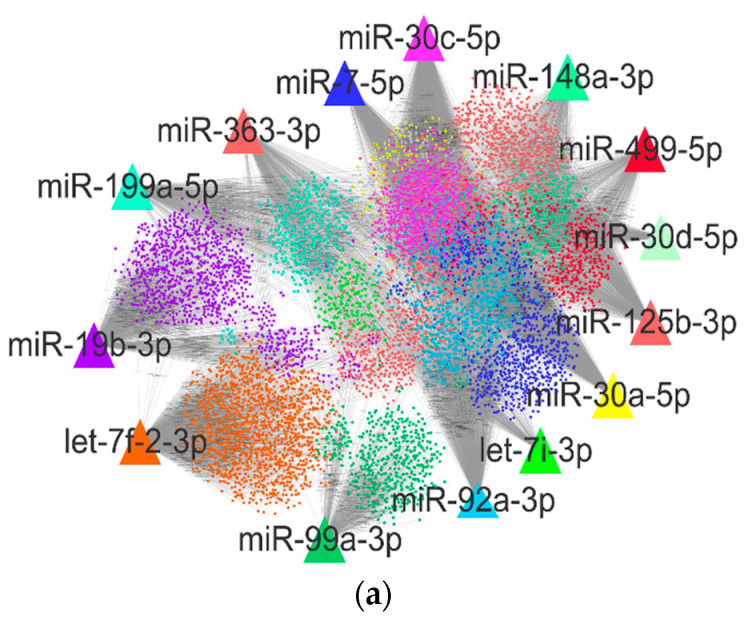

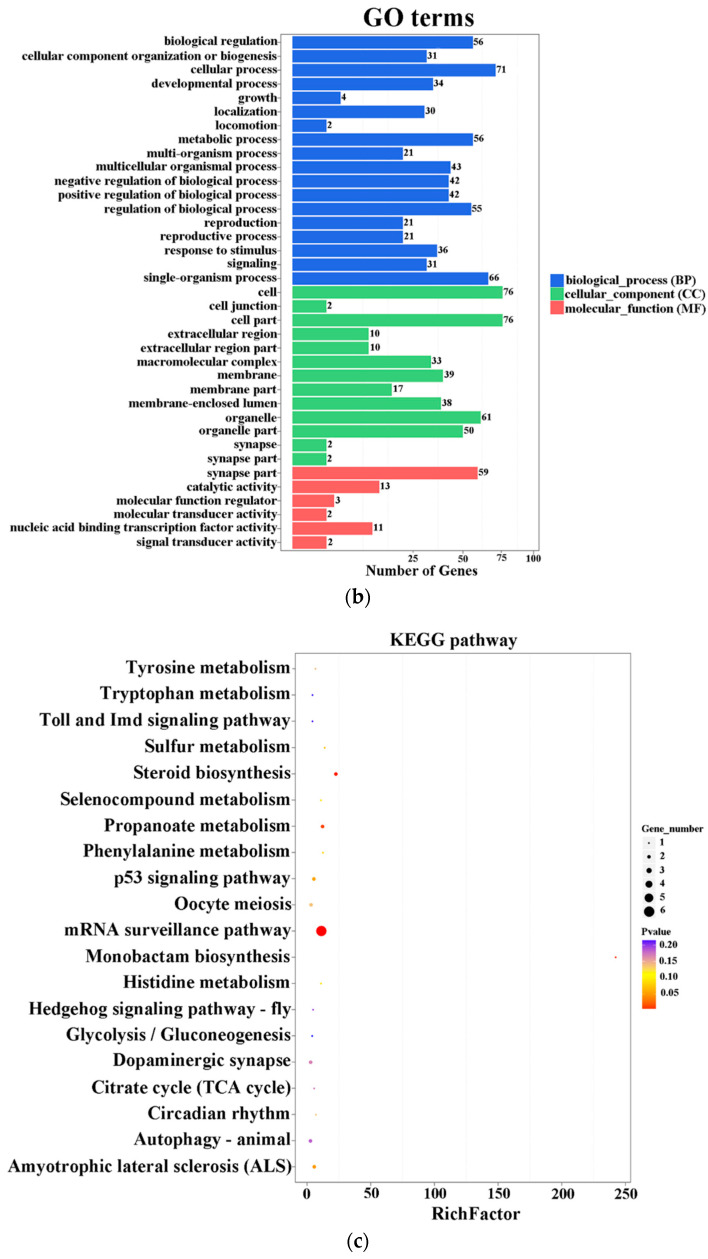
Functional analysis of target genes of DEMs. (**a**) DEMs target gene network associated with development and metabolism of skeletal muscle in rabbits. Colors represent target gene groups regulated by seven DEMs. (**b**) Enrichment analysis of target gene Gene Ontology (GO) functions and (**c**) Kyoto Encyclopedia of Genes and Genomes (KEGG) signaling pathways from DEMs in skeletal muscle from CON–G and HFD–G rabbits.

A total of 6739 potential target genes were obtained for 14 DEMs through Targetscan, miRanda and RNAhybrid target-gene prediction analyses, and the resulting miRNA target gene network is shown in Figure 3a. All target genes were submitted to the DAVID database to conduct Gene Ontology (GO) and Kyoto Encyclopedia of Genes and Genomes (KEGG) pathway enrichment analyses of functional differences between DEMs in skeletal muscle from CON–G and HFD–G rabbits. The GO enrichment analysis showed that the target genes were involved in 347 GO terms (*p* < 0.05), 190 GO terms in biological processes (BP), 90 GO terms in cellular components (CC), and 67 GO terms in molecular functions (MF). The top GO terms in each category are shown in Figure 3b. The BP GO terms were mostly involved in the downregulation of transcription from RNA polymerase II promoter, small GTPase mediated signal transduction, and intracellular signal transduction functions. The CC GO terms were mainly involved in nucleoplasm, Golgi apparatus and cytoplasm functions. The MF GO terms were primarily involved in transcriptional activator activities, sequence-specific RNA polymerase II core promoter proximal region binding, and protein serine and ATP binding functions. The KEGG enrichment analysis of DEM target genes indicated that they were involved in 115 pathways (*p* < 0.05). The top 20 KEGG pathways were mainly involved in MAPK signaling, endocytosis, regulation of actin cytoskeleton, circadian entrainment and PI3K-Akt signaling (Figure 3c). Enrichment analysis of target genes showed that these miRNAs may play an important role in regulating the development and metabolism of rabbit muscle.

### 2.4. Transcriptome Analysis

**Figure 4:**
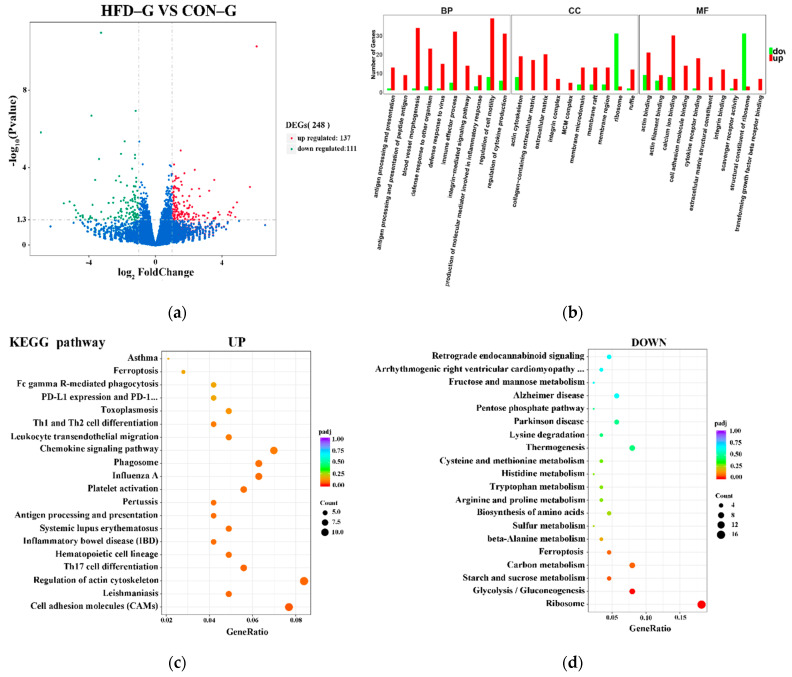
DEGs analysis of transcriptome between CON–G and HFD–G. (**a**) Volcano map of differentially expressed genes for transcriptome sequencing. Green, blue, and red represent downregulated genes with significant differences, genes with nonsignificant differences, and upregulated genes with significant differences, respectively. (**b**) Enrichment analysis diagram of GO function. Green and red represents downregulated and upregulated differentially expressed genes, respectively. (**c**,**d**) KEGG signaling pathway of upregulated and downregulated genes. Color of scattered dots varies from blue to red, representing a range of nonsignificant to significant differences, and circle size indicates number of enriched differentially expressed genes.

Figure 4a shows a volcano plot of the 248 differentially expressed genes (DEGs) (137 upregulated and 111 downregulated) identified from a total of 18,862 genes with the |log2FoldChange| > 1 and *p* < 0.05 screening criteria. The specific difference information is shown in Table S2. All DEGs were submitted to the DAVID database to run GO term and KEGG pathway enrichment analysis. The GO analysis indicated that these DEGs mainly downregulate ribosome and structural constituents of ribosomes and upregulate blood vessel morphogenesis, immune effector processes, cell motility, cytokine production, and calcium ion binding functions (Figure 4b). The KEGG pathway enrichment analysis (Figure 4c,d) uncovered upregulated DEGs that were significantly enriched in inflammatory signaling pathways (Th17 cell differentiation, antigen processing and presentation, chemokine signaling, Th1 and Th2 cell differentiation) and downregulated DEGs that were significantly enriched in amino acid metabolism and synthesis pathways (glycolysis/gluconeogenesis, starch and sucrose metabolism, carbon metabolism, biosynthesis of amino acids). The results of differential gene enrichment indicate that obesity could increase the intramuscular inflammatory response in rabbits.

### 2.5. Identification and Functional Analysis of Differentially Expressed Proteins (DEPs) 

**Figure 5:**
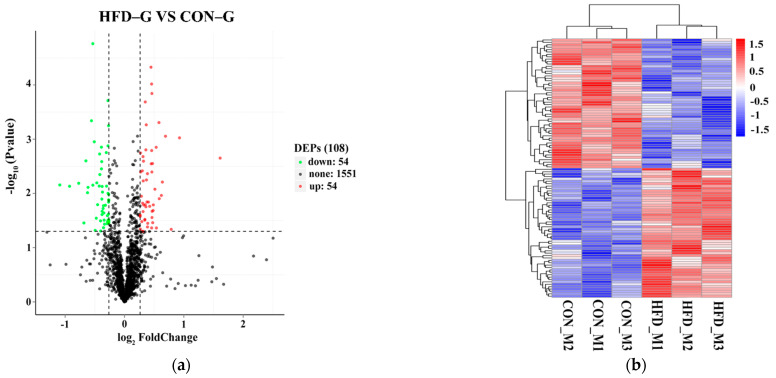

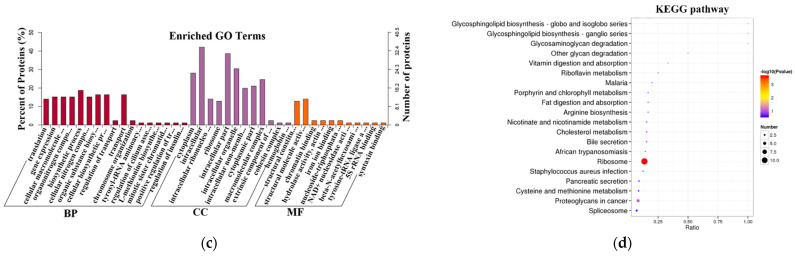
Screening and functional enrichment analysis of GO functions and KEGG signaling pathways of differentially expressed proteins in skeletal muscle samples from CON–G and HFD–G rabbits. (**a**) Colors in volcano map represent downregulated proteins (green dots), upregulated proteins (red dots), and nonsignificant proteins (black dots). (**b**) Colors in the heatmap represent changes in protein levels, from high (red) to low (blue). Left brackets represent clustering relationships among genes, and top brackets represent clustering relationships among samples. (**c**) In GO functional enrichment diagram, three colors represent biological processes (BP), cellular components (CC), and molecular functions (MF). Height of each column represents number of enriched genes. (**d**) In KEGG pathway enrichment diagram, color ranges from red (significant) to blue (nonsignificant), and circle size indicates number of enriched genes.

A total of 286,536 spectra and 39,425 peptide spectrum matches (PSMs) were identified using a tandem mass tag spectrometry analysis with Proteome Discoverer 2.2 software. FDR verification (FDR > 1%) determined that 14,075 peptides and 2079 proteins were reliable proteins. The t–test of relative quantitative values of DEP proteins indicated that 1659 proteins were significantly differentially expressed in skeletal muscle from CON–G and HFD–G rabbits (Figure 5a). A heat map of differentially expressed proteins revealed that there were 108 DEPs (54 upregulated and 54 downregulated) in skeletal muscle samples from CON–G and HFD–G rabbits (Figure 5b). 

GO function and KEGG pathway enrichment analysis of DEPs was performed to increase our understanding of the biological functions of skeletal muscle DEPs from CON–G and HFD–G rabbits (Figure 5c,d). GO function enrichment analysis indicated that DEPs in rabbit skeletal muscle were mainly concentrated on BP GO terms (translation, gene expression, cellular macromolecule biosynthetic processes), CC GO terms (cytoplasm, intracellular, ribosomes), and MF GO terms (structural constituents of ribosomes, structural molecular activities, chromatin binding). Meanwhile, KEGG pathway enrichment analysis indicated that the significant pathways involving DEPs in rabbit skeletal muscle included ribosome, glycosaminoglycan degradation, glycosphingolipid biosynthesis-globo and isoglobo series, and glycosphingolipid biosynthesis-ganglio series. Thus, the DEPs in rabbit skeletal muscle are involved in intracellular changes in gene transcription, translation, and ribosomal protein structure. The results of differential protein function enrichment indicate that obesity may increase carbohydrate metabolism and synthesis in rabbit muscle.

### 2.6. Integrated Analysis of DEGs and DEPs 

**Figure 6:**
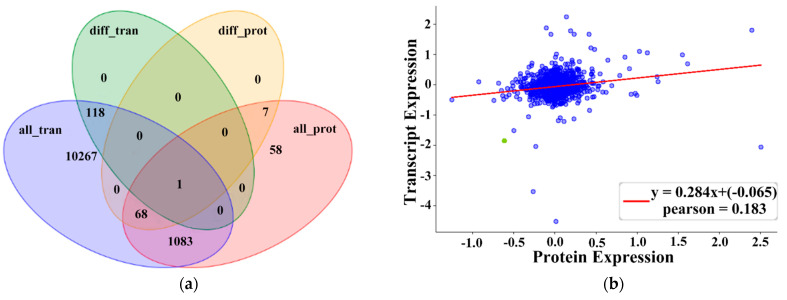
Venn diagram of all differentially transcribed genes and differentially translated proteins and correlation analysis of transcriptome and proteome expression levels in skeletal muscle samples from CON–G and HFD–G rabbits. (**a**) In Venn diagram, blue (all_tran) represents all genes obtained from transcriptome, green (diff_tran) represents differentially expressed genes identified by the transcriptome, red (all_prot) represents all proteins identified by the proteome, and orange (diff_prot) represents differentially expressed proteins identified by the proteome. (**b**) In correlation analysis graph, green dot represents significant differentially expressed protein and blue dot represents nonsignificant differentially expressed protein. Abscissa represents differentially translated proteins (log_2_FC) and ordinate represents differentially transcribed genes (log_2_FC) in skeletal muscle samples from CON–G and HFD–G rabbits.

A total of 18,863 genes and 1659 proteins were identified in RNA-seq and TMT analyses. A Venn diagram of DEGs and DEPs in skeletal muscle samples from CON–G and HFD–G rabbits shows a total of 1152 differentially expressed genes, of which 60 are significantly different (Figure 6a). The correlation between transcriptome and proteome expression levels in skeletal muscle from CON–G and HFD–G rabbits is shown in Figure 6b. Subsequently, the correlated DEGs and DEPs were submitted to the DAVID database to perform GO term and KEGG pathway enrichment analysis to identify genes associated with skeletal muscle metabolism (Table 3). Seven DEGs (*CRYL1*, *VDAC3*, *BST1*, *APIP*, *ENOPH1*, *SLC37A4*, and *GSTO1*) were downregulated, three DEPs (GPT2, FLOT2, and L2HGDH) were downregulated, and two DEGs (*AQP4* and *TXNDC12*) were upregulated in five metabolism-related pathways (cysteine and methionine metabolism, glutathione metabolism, apelin signaling, nicotinate and nicotinamide metabolism, and insulin signaling). The above combined analysis results indicate significant differences in the transcription and translation results of different genes, but the correlative genes and proteins caused by obesity merit future studies on the muscle metabolism of rabbits.

### 2.7. Network Analysis of DEMs, DEGs, and DEPs

**Figure 7:**
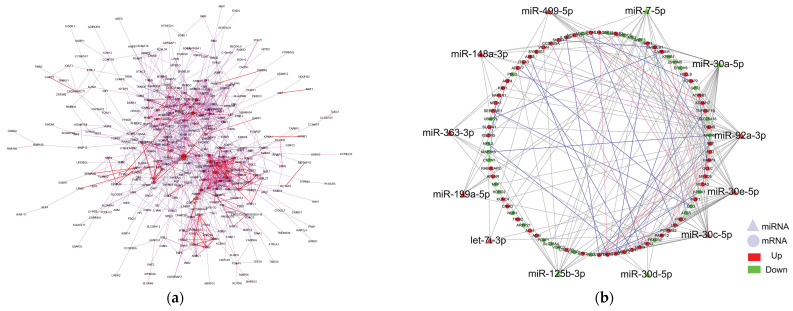
Combined interaction network diagram of DEGs and DEPs and regulatory network diagram of key miRNAs. (**a**) In protein interaction network diagram, circles range in color from blue to purple to red, representing the greater role of genes in the network, and each circle represents a gene. Larger circles represent a greater role of genes. Lines from purple to red show correlations between genes from low to high. (**b**) In miRNA network regulation diagram, triangles represent miRNAs, and red and blue represent upregulation and downregulation, respectively. Color of lines in the network goes from red to blue to indicate that correlation goes from high to low.

To better understand the regulatory relationship between genes and proteins, combined interaction network analysis was performed to increase our understanding of the regulation of DEGs and DEPs in rabbit skeletal muscle using STRING online software. The construction of the combined interaction network was completed by importing the correlation coefficient from STRING into Cytoscape 3.7.0 software (Figure 7a). Combined interaction network and miRNA target gene network analysis predicted that 12 miRNAs (miR-7-5p, miR-499-5p, miR-125b-3p, miR-30d-5p, miR-30e-5p, miR-363-3p, miR-92a-3p, let-7i-3p, miR-30a-5p, miR-199a-5p, miR-30c-5p, and miR-148a-3p) regulated 85 genes (65 DEGs and 20 DEPs; Figure 7b). Lastly, miRNA–mRNA network analysis revealed that seven miRNAs–mRNA pairs play a key role in the regulation of seven genes (*MAP3K3*, *MYH9*, *PARP12*, *GPT2*, *VDAC3*, *NCAM1*, and *GCLC*) and three transcription factors (MYBL2, STAT1 and IKZF1) involved in rabbit skeletal muscle metabolism (Table 4). All of these important regulatory networks of DEMs, DEGs, and DEPs in rabbits with induced by obesity enable us to better understand the potential molecular mechanism of muscle metabolism regulation.

## 3. Discussion

High-fat and high-sugar diets are important factors causing obesity in human beings. These types of diets are essential for the study of metabolic differences among animals [18]. Compared with different types of induction diets (unsaturated fatty acids, protein, and saccharides), a high–fat diet is more likely to increase the amount of fat deposition in different tissues of animals, thereby increasing the incidence of obesity-related metabolic diseases [19]. Especially, the amount of intramuscular fat deposition can influence the oxidative metabolic capacity and insulin resistance of skeletal muscle [20]. Studies in rodents and other animals have also shown that intramuscular fat deposition and the proportion of unsaturated fatty acids are closely related to body fat consumption [21,22]. Similarly, a high–fat diet will also lead to increased intramuscular fat content in humans, which will lead to increased insulin resistance and inflammatory factors [23]. There are great differences in intramuscular fat deposition in different animals, which is related to the metabolic mechanism of muscle. In view of this, it is necessary to explore the potential metabolic mechanism of rabbit skeletal muscle. The present study was aimed at screening out the DEMs, DEGs, and DEPs in rabbit skeletal muscle from two groups (HFD–G and CON–G). Additionally, functional analysis of DEM–DEG pairs and regulatory network analysis of DEMs, DEGs, and DEPs associated with the development and metabolism of skeletal muscle in rabbits were conducted. 

microRNAs can be treated as key regulatory factors for a variety of important target genes involved in protein coding that influence a variety of phenotypes. Mature miRNA sequences are classes of 22 nt short non-coding RNAs processed in the cytoplasm by nuclear endogenous transcripts (pri-miRNAs). A comparison of miRNAs in various organisms by genome sequences revealed that they evolved in a different way from genes. The gene family of miRNAs evolved in a continuously updating way, in which new miRNAs were generated in each derived evolutionary pedigree [24]. Based on the analysis of existing miRNA data, it appears that some miRNAs in transcriptional binding sites of the 3′ non-coding regions of target genes are conserved, suggesting the potential involvement of miRNAs in animal evolution [25]. For example, the let-7 family, miR-9, and miR-183 may have similar developmental regulatory functions across model organisms [26,27,28]. The evolutionary model of miRNAs also fully demonstrates that they have complex regulatory roles in cell development and regulation.

In this study, small RNA sequences showed that known miRNAs were highly expressed in obese rabbits. However, the expression levels of novel miRNA were lower than those of known miRNAs. Low miRNA expression plays a certain role in the regulation of cells biological responses [29]. Conversely, high miRNA expression plays a key roles in the regulation of targeted molecules in tissues and cells, indicating that miRNAs are highly involved in intracellular molecular regulation [30]. Highly expressed miRNAs that are highly conserved have more abundant and extensive functional mechanisms associated with intracellular gene regulation. We identified 520 novel miRNAs in rabbit skeletal muscle that may potentially have regulatory effects on muscle metabolism and need further verification. Notably, among the 20 miRNA identified, there were four from the miR-30 family (miR-30e-5p, miR-30c-5p, miR-30a-5p, and miR-30d-5p) and two from the let family (let-7i-3p and let-7f-2-3p), two families known to be important for skeletal muscle metabolism [31,32]. miR-30-5p inhibits muscle cell differentiation and regulates the alternative splicing of *Trim55* and *INSR* by targeting *MBNL* [33]. The miR-30 family also regulates myogenic differentiation and targets the *Tnrc6a* gene to downregulate the miRNA pathway, indicating that the miR-30 family is a key factor in muscle development [34]. Additionally, miR-148a-3p, miR-499-5p, miR-199a-5p, miR-133-3p, and miR-92a-3p were associated with myofiber specification, apoptosis, and the proliferation of skeletal muscle cells [35,36,37]. Combined miRNA–target gene prediction and functional enrichment analysis indicated that the top 10 KEGG pathways were involved in six metabolic signaling pathways (MAPK signaling, cGMP–PKG, insulin, PI3K–Akt, cAMP, and calcium signaling) associated with skeletal muscle metabolism in rabbits. The miRNA–target gene enriched pathways reflected a possible function of DEMs in the regulation of skeletal muscle development. These results indicate that the role of the miR-30 family and other miRNAs in rabbit skeletal muscle development and metabolism merit further study. 

Transcriptome analysis demonstrated that upregulated pathways were closely associated with inflammatory signaling pathways. Previous studies have shown that a high–fat diet induces obesity by increasing intramuscular fat content, triggering a skeletal muscle inflammatory response [38]. The TLR4 signaling pathway and other pattern recognition receptors (PRRS) in muscle cells can respond to inflammatory signals and influence metabolic changes [39]. The T cells and macrophages are involved in the production of inflammatory cytokines and insulin resistance in skeletal muscle [40,41]. Similarly, we found that Th17 cell differentiation, antigen processing and presentation, and chemokine signaling pathways were upregulated in the skeletal muscle of obese rabbits in the HFD-G group. Additionally, glycolysis/gluconeogenesis pathways play an essential role in the process of carbohydrate metabolism in various tissues of the body. The disorder of this pathway can cause the occurrence of human myopathy and is closely associated with function of adipose tissue macrophages (ATMs) in a high–fat diet–induced model of mice for responding to cellular insulin resistance [42,43]. The Gene Expression Omnibus (GEO) database of humans from non-insulin dependent diabetes mellitus (NIDDM) and obesity found that the biosynthesis of amino acids and notch signaling pathway were down regulated in adipose tissues [44]. Our results indicated that the glycolysis/gluconeogenesis pathway and amino acid synthesis pathway were down regulated in skeletal muscle of obese rabbits, which were similarly consistent with these previous studies. However, the metabolic signal pathways in this study (glycosaminoglycan biosynthesis-chondroitin sulfate/dermatan sulfate (*CSGALNACT1* and *CHSY3*), adipocytokine signaling (*TNFRSF1B*, *mTOR*, *ACSL5* and *ADIPOR2*), gastric acid secretion (*ADCY7*, *SLC9A1* and *ATP1A1*)) seemed to not play a key role in the regulation of skeletal muscle metabolism. 

Glycosaminoglycan (GAG) is a type of polysaccharide with a long-chain macromolecule that is widely present in the extracellular matrix of vertebrate tissues. There are six types of GAGs, including hyaluronic acid (HA), chondroitin sulfate (CS), dermatan sulfate (DS), keratan sulfate (Kep-S), and heparin (Hep) [45]. GAGs have been shown to be involved in many physiological and pathological processes, and they can also be used as effective anti-inflammatory drugs for the treatment of diseases [46]. Glycosphingolipids are a class of lipid compounds containing glycosyl ligands. They are an indispensable part of the cell membranes that can be synthesized or catabolized with sphingomyelin to be transformed into ceramides, which affect cellular immune response, development, recognition, and differentiation [47]. Human obesity studies have found that saturated fatty acids can induce TLR4 expression to activate MyD88- and TRIF-dependent pathways that participate in disease occurrence, thereby increasing the synthesis of ceramides in skeletal muscle and liver tissues [48,49], which is crucial for skeletal muscle to improve the symptoms of insulin resistance caused by obesity [50]. The proteomics enrichment analysis in this study confirmed that the glycosaminoglycan degradation and glycosphingolipid biosynthesis pathways were significant and could perhaps play a key role in the regulation of skeletal muscle metabolism.

The integrated analysis of DEG and DEP changes indicated that a high–fat diet induced the differential expression of genes associated with mitochondrial proteins, mitochondrial oxidative metabolism factors, and gluconeogenesis of skeletal muscle in rabbits. Mitochondria play a vital role in the regulation of energy metabolism in skeletal muscle. Oxidative damage to the mitochondria of skeletal muscle can cause insulin resistance [51]. Mitochondrial proteins are closely associated with energy metabolism in skeletal muscle. VDAC3 is a member of the voltage-dependent anion channel (VDACs) protein family, also known as mitochondrial porins, and plays a vital role in transporting mitochondrial metabolites [52]. Glutamate pyruvate transaminase 2 (GPT2) protein, also known as alanine transaminase 2 (ALT2) or alanine aminotransferase 2 (ALAT2), is located in mitochondria and involved in amino acid metabolism and the tricarboxylic acid (TCA) cycle. A change in the GPT2 protein can lead to metabolic disease [53]. Expression of VDAC3 and GPT2 proteins was significantly downregulated in the skeletal muscle of obese rabbits in this study. The accumulation of fatty acids in skeletal muscle can lead to abnormal mitochondrial oxidative metabolism. The glutathione S-transferase (GST) enzyme is widely present in various tissues. Its main function is to eliminate free radicals and detoxify the body, and it plays a critical role as an antioxidant and in the detoxification metabolism of poisons. The overexpression of the glutathione S transferase omega1 (GSTO1) enzyme can enhance antioxidant stress in animals [54]. Enolase phosphatase 1 (ENOPH1) is an important enzyme in the methionine rescue pathway. It has a phosphatase function and belongs to the dual function enzymes of atypical enolase activity. It plays an important role in regulating oxidative stress response [55]. Thioredoxin domain-containing protein 12 (TXNDC12) is highly expressed in liver, brain, and skeletal muscle tissues, and has a function in response to cell oxidative stress and cellular detoxification [56]. We also found that the proteins GSTO1, ENOPH1, and TXNDC12 had significant differential expressions in the skeletal muscle of rabbits in the obese group (HFD-G) versus rabbits in the control group (CON-G).

Other relevant skeletal muscle proteins include SLC37A4, known as glucose 6-phosphate translocase (G6PT), which is closely associated with glycogen storage diseases [57]. The G6PT/G6pase-complex is widely expressed in many tissues, and the G6pase-complex, expressed in gluconeogenic tissues, maintains glucose homeostasis and neutrophil/macrophage energy homeostasis and function [58]. The *BST1* gene encodes a multifunctional extracellular enzyme involved in regulating adenosine diphosateribose (ADPR). As an intercellular receptor, *BST1* is involved in inflammatory response by changing cell morphology, intercellular adhesion ability, and cell migration to regulate leukocyte function [59]. We found that the *SLC37A4* and *BST1* genes were significantly overexpressed in skeletal muscle, suggesting the existence of a disorder in the gluconeogenic pathway of skeletal muscle in obese rabbits (HFD-G). However, most genes had inconsistent transcription and protein levels, suggesting that the regulation process of mRNA translation events plays a key role in the response to metabolic changes in the skeletal muscle of obese rabbits. Previous studies showed that gene transcription levels are not completely correlated with protein translation levels, indicating that the regulation of mRNA translation events is a complex process [60]. Network analysis of DEMs, DEGs, and DEPs identified seven miRNA–mRNA pairs involved in cell metabolism, muscle development, and disease. We also found three transcription factors (MYBL2, STAT1 and IKZF1) regulated by two miRNAs (miR-92a-3p and miR-30a/c/d-5p) that may play a key role in the regulation of skeletal muscle metabolism. Transcription factors play an important regulatory role in gene transcription by recognizing transcription binding sites in the promoter region of genes in response to complex external stimuli. Previous studies have shown that these three transcription factors were mainly associated with many process of cell cycle regulation, aging, carcinogenesis, and tumorigenesis, through a variety of metabolic signaling pathways, such as Chemical signaling pathway, JAK-STAT signaling pathway, and MAPK signaling pathway [61,62,63]. In this study, we found that they were closely involved in the skeletal muscle metabolism process of obese rabbits induced by high–fat diet, which were worthy of being used as important regulators for the future study of human obesity syndrome.

## 4. Materials and Methods

### 4.1. Construction of the Obesity Model with Young Rabbits

Female Tianfu black rabbits (*n* = 16) were selected from the teaching and research rabbit farm of Sichuan Agriculture University. All rabbits were kept under the same management conditions and were regularly vaccinated. The 16 weaned rabbits were divided into 2 groups at about 35 days of age, a control group (CON–G; *n* = 8) fed a commercial diet, and an obese group (HFD–G; *n* = 8) fed a high–fat diet composed of a commercial diet mixed with 10% pork lard. Rabbits in both groups were fed from 35 to 70 days of age. The methods in this study were based on our previous research [17], which indicated that body weight at 70 days of age (2–2.5 kg) is an important reference index to evaluate obesity in rabbits under the same feeding conditions. In this study, 3 rabbits with the highest body weight in each group were screened out before all of the rabbits were killed by intravenous injection. The 3 rabbits sampled from each group (CON–G and HFD–G) were used to collect samples of the right *Biceps femoris* muscle after slaughter at 70 days of age. Muscle samples were rapidly stored in liquid nitrogen at −80 °C.

### 4.2. Total RNA Extraction and Small RNA Sequencing

Total RNA was extracted from the skeletal muscle samples (stored at −80 °C) according to the TakaRa MiniBEST Universal RNA Extraction Kit instruction manual (TakaRa, Japan). RNA concentration and purity were determined by using a NanoDrop 2000 spectrophotometer (Thermo Fisher Scientific, Wilmington, DE, USA). Small RNA was sequenced using SE50 sequencing mode. The sequenced reads were filtered by SOAPnuke software (https://github.com/BGI-flexlab/SOAPnuke/releases/tag/SOAPnuke2.1.5, accessed on 15 July 2020). A comparison of sRNA tags (tag ≥ 18 nt) with the species genome and an evaluation of all the comparison data and sample distributions were performed with Bowtie2 software (http://bowtie-bio.sourceforge.net/bowtie2/index.shtml, accessed on 20 July 2020). All known miRNAs were identified by using the miRBase database (http://www.mirbase.org/, accessed on 20 July 2020). Novel miRNAs were predicted from unannotated sRNA by using MIREAP (https://tools4mirs.org/software/sequencing_analysis/mireap/, accessed on 20 July 2020) and miRNA visualization software (https://tools4mirs.org/software/sequencing_analysis/mireap/, accessed on 20 July 2020). The edgeR package (https://bioconductor.org/packages/release/bioc/html/edgeR.html, accessed on 20 July 2020) was used to analyze the differential expression of miRNAs. Differentially expressed miRNAs were filtered and identified as significant using the standard log2fold change (log_2_FC) and false discovery rate (FDR), with thresholds of |log_2_FC| ≥ 1 and FDR < 0.05 for differentially expressed miRNAs. The target genes were predicted by Targetscan (http://www.targetscan.org/vert_72/, accessed on 20 July 2020), miRanda (https://tools4mirs.org/software/target_prediction/miranda/, accessed on 20 July 2020), and RNAhybrid (https://bibiserv.cebitec.uni-bielefeld.de/rnahybrid, accessed on 20 July 2020) software.

### 4.3. Transcriptome. Sequencing (RNA-seq)

Sequencing libraries were generated using NEBNext^®^ UltraTM RNA Library Prep Kit for illumina^®^ (NEB, Ipswich, MA, USA) following the manufacturer’s recommendations. The library preparations were sequenced on an illumina Novaseq platform (https://www.illumina.com/systems/sequencing-platforms/novaseq.html, accessed on 15 August 2020). After screening the total_reads data for high quality clean reads, HISAT2 v2.0.5 software (http://daehwankimlab.github.io/hisat2/, accessed on 15 August 2020) was used to match clean reads to the reference genome sequence (GCF_000003625.3) to assess the overall sequencing quality. FeatureCounts v1.5.0-p3 (https://academic.oup.com/bioinformatics/article/30/7/923/232889, accessed on 15 August 2020) was used to count the number of reads mapped to each gene. Subsequently, the fragments per kilobase of transcript per million mapped reads (FPKM) for each gene was calculated based on the length of the gene and reads count mapped to this gene. Information from differentially expressed genes (DEGs) from the RNA-seq analysis was filtered with the edgeR (3.18.1) and DESeq2 (1.16.1) (https://bioconductor.org/packages/release/bioc/html/DESeq2.html, accessed on 15 August 2020) R packages. Significantly differentially expressed genes were screened out according to their threshold log_2_FC and *p* values (|log_2_FC| ≥ 1 and *p* < 0.05).

### 4.4. Protein Isolation, Enzymolysis, and TMT Labeling

Total proteins in muscle samples were extracted using a mammalian protein extraction kit (Product ID C600589, Sangon Bioengineering Co., Ltd., Shanghai, China) according to the manufacturer’s instructions. Protein concentrations were detected using a Bradford protein quantitative kit (Product ID C503031-1000, Sangon Bioengineering Co., Ltd., Shanghai, China) following the manufacturer’s instructions. A bovine serum albumin (BSA) standard protein solution was prepared with a gradient concentration ranging from 0 to 0.5 g/L. The protein concentration in samples was calculated using a standard curve constructed using the absorbance of the BSA standard protein solution. Then, 120 μg of each protein sample was mixed and digested with trypsin and CaCl_2_ at 37 °C overnight. After elution (0.1% formic acid, 3% acetonitrile, and 70% acetonitrile), protein peptides from the protein hydrolysates were labeled by using acetonitrile-soluble tandem mass tagging kits and reagents (Thermo Fisher Scientific, Wilmington, DE, USA).

### 4.5. LC-MS/MS Analysis

After desalting, the lyophilized products were separated by distillation using an L-3000 HPLC system with a Waters BEH C18 chromatographic column (4.6 × 250 mm, 5 μm) and a column temperature of 50 °C. A 1 μg supernatant of each distillate was assessed for liquid quality detection with an EASY-nLCTM 1200 nano-flow UHPLC system (Thermo Fisher Scientific, Wilmington, DE, USA). The raw data were generated with a Q ExactiveTM HF-X mass spectrometer (Thermo Fisher Scientific, Wilmington, DE, USA) and a Nanospray Flex™ (SI) ion source with data-dependent acquisition mode.

### 4.6. Database Search and Protein Identification and Quantification

Proteome Discoverer 2.2 software (https://www.thermofisher.com/us/en/home/industrial/mass-spectrometry/liquid-chromatography-mass-spectrometry-lc-ms/lc-ms-software/multi-omics-data-analysis/proteome-discoverer-software.html, accessed on 25 August 2020) was used to search the Ensemble database (Oryctolagus_cuniculus_41055_release 100_ensembl.fa) and quantify the peptide data to filter and retain peptide spectrum matches (PSMs) with more than 99% confidence. Credible PSMs were verified with FDR, and peptides and proteins with FDR > 1% were removed. A t–test was used to compare the protein data from the control (CON–G) and obese (HFD–G) rabbits. Proteins with significant differences (*p* < 0.05, |log_2_FC| > 0 (ratio > 1.2 or ratio < 0.83) were defined as differentially expressed proteins (DEPs).

### 4.7. GO and KEGG Enrichment Function Analysis of Target Genes, DEGs, and DEPs

The miRNA target genes, DEGs in RNA-seq, and DEPs were classified using GO and KEGG with DAVID online software (https://david.ncifcrf.gov/, accessed on 20 October 2020). GO terms and KEGG pathways with corrected *p*-values < 0.05 were considered significantly enriched. The diagram R package (https://cran.r-project.org/web/packages/diagram/vignettes/diagram.pdf, accessed on 20 October 2020) and GraphPad Prism 8 (https://www.graphpad.com/scientific-software/prism/, accessed on 20 October 2020) were used to draw diagrams.

## 5. Conclusions

We constructed a high–fat–diet–induced rabbit obesity model and detected differentially expressed miRNAs, mRNAs, and proteins, which were all significantly enriched in metabolic pathways of amino acids, glycosaminoglycan, and glycosphingolipid in skeletal muscle. We developed a reliable regulatory network of differentially expressed genes and proteins involved in the metabolism of skeletal muscle in rabbits using an integrated analysis of DEMs, DEGs, and DEPs. We also found a number of statistically significant interactions between miRNAs and mRNAs as well as three key transcription factors (MYBL2, STAT1, and IKZF1) that were regulated by two types of miRNAs (miR-92a-3p and miR-30a/c/d-5p). These results enhance our understanding of molecular mechanisms associated with rabbit skeletal muscle growth and metabolism and provide a basis for future studies in the metabolic diseases of human obesity.

## Figures and Tables

**Table 1:** Quality statistics for data generated from small RNA deep sequencing.

Sample	Clean Reads	Clean Bases	Q20(%) ^1^	GC(%) ^2^
CON–M1	10,342,003	228,333,247	99.85	46.81
CON–M2	10,354,071	230,699,706	99.58	45.86
CON–M3	10,268,517	226,301,819	99.6	45.47
HFD–M1	10,097,828	223,290,399	99.57	46.89
HFD–M2	10,314,308	228,316,610	99.85	46.54
HFD–M3	10,293,639	228,541,211	99.57	45.57

^1^ Q20(%) = quality score percentage (–10log_10_(e) × 100) = probability percentage of 1 incorrect in 100 base calls; ^2^ GC(%) = percentage of (G + C) of all bases (A + T+ G +C).

**Table 2:** Differential expression information of top 10 upregulated and top 10 downregulated DEMs between CON–G and HFD–G.

Gene ID	CON-G Mean	HFD-G Mean	log_2_FC	*p*-Value	Regulation
miR-499-5p	220.6667	19,848	6.435477	7.98 × 10^−8^	Up
miR-30e-5p	708	12,837.67	4.043857	0.000164	Up
miR-363-3p	248.6667	3624.333	3.755788	0.000396	Up
let-7i-3p	10857.33	103,267.7	3.202507	0.001939	Up
miR-19b-3p	26.66667	255	3.170493	0.002356	Up
miR-26c	28,567	260,880.7	3.065703	0.002842	Up
miR-199a-5p	1258.666667	20,851	3.857928946	0.000287665	Up
miR-148a-3p	8216	55,153	2.715061	0.007331	Up
miR-30c-5p	1221.667	6581.333	2.313511	0.020359	Up
miR-92a-3p	464.6667	1771.333	2.019203	0.040922	Up
miR-30a-5p	18,928	5391	−1.94918	0.047754	Down
miR-30d-5p	61,709.33	16,716	−2.00517	0.042075	Down
miR-125b-3p	22,741.33	5481.667	−2.15275	0.029903	Down
miR-7	3289.667	652.6667	−2.47901	0.013516	Down
miR-99a-3p	129,929	23,586	−2.50911	0.012485	Down
miR-3596	25,770.33	3702.667	−2.82627	0.005459	Down
let-7f-2-3p	22,724.67	3421	−2.85288	0.005083	Down
miR-218b	471.3333	65.33333	−2.92167	0.004402	Down
miR-20a-2-3p	360.3333	42.33333	−3.09849	0.002779	Down
miR-133-3p	182,602.7	18,092	−3.29051	0.001512	Down

**Table 3:** GO function, KEGG signaling pathway, and log_2_FC for key differentially expressed genes and proteins involved in rabbit skeletal muscle metabolism.

Gene ID	GO Function	KEGG Signaling Pathway	Transcribed Genes (log_2_FC)	Translated Proteins (log_2_FC)
CRYL1	Primary metabolic process	Pentose and glucuronate interconversions (map00040)	−0.34786	−0.2887
AQP4	Transport	Bile secretion (map04976)	−0.41118	0.458027
VDAC3	Intracellular	Cholesterol metabolism (map04979)	−0.4851	−0.62335
BST1	Hydrolase activity, acting on glycosyl bonds	Nicotinate and nicotinamide metabolism (map00760)	−0.17229	−0.34353
APIP	intracellular	Cysteine and methionine metabolism (map00270)	−0.40076	−0.46668
ENOPH1	-	Cysteine and methionine metabolism (map00270)	−0.13566	−0.30252
TXNDC12	Regulation of biological quality	Glutathione metabolism (map00480)	0.049503	0.349659
FLOT2	-	Insulin signaling pathway (map04910)	0.126643	−0.26827
SLC37A4	Transport	Carbohydrate digestion and absorption (map04973)	−0.33128	−0.2698
GSTO1	Intracellular	Glutathione metabolism (map00480)	−0.28975	−0.26433
L2HGDH	-	Butanoate metabolism (map00650)	0.17351	−0.47531
GPT2	Biosynthetic process	2-Oxocarboxylic acid metabolism (map01210)	0.208941	−0.26321

**Table 4:** Description of regulatory networks for seven key miRNAs–mRNA pairs involved in rabbit skeletal muscle metabolism.

Gene ID	Transcribed Genes (log2FC)	Translated Proteins (log2FC)	miRNA Regulation	Gene Description	Transcription Factor
MAP3K3	0.22279	−0.35343	let-7i-3p	Mitogen-activated protein kinase kinase kinase 3	-
MYH9	0.539348	0.143813	miR-92a-3p,miR-363-3p	Myosin heavy chain 9	-
PARP12	0.74067	-	let-7i-3p	Poly(ADP-ribose) polymerase family member 12	-
GPT2	0.208941	-0.26321	miR-30a-5p,miR-30c-5p	Glutamic--pyruvic transaminase 2	-
VDAC3	−0.4851	−0.62335	miR-7-5p	Voltage dependent anion channel 3	-
NCAM1	0.593775	-	miR-30d-5p,miR-30a-5p	Neural cell adhesion molecule 1	-
GCLC	0.449528	−0.16912	miR-30a-5p	Glutamate-cysteine ligase catalytic subunit	-
MYBL2	1.563326	-	miR-30c-5p,miR-30a-5p,miR-30d-5p	MYB proto-oncogene like 2	MYB
STAT1	0.849922	0.078617	miR-30c-5p	Signal transducer and activator of transcription 1	STAT
IKZF1	1.978475	-	miR-30c-5p,miR-92a-3p	IKAROS family zinc finger 1	zf-C2H2

## Data Availability

This paper utilized original data not used in other publications. Although data sharing is not applicable to this article, a permission request to upload the original data to a public database could be processed.

## Supplementary Materials

The following are available online at https://www.mdpi.com/article/10.3390/ijms22084204/s1, Figure S1: Anatomical differences of subcutaneous fat (SF) and visceral fat (VF) in rabbit between CON-G (a) and HFD-G (b), Table S1: The different expressed information of novel miRNAs between CON–G and HFD–G, Table S2: The different expressed information of the genes between CON–G and HFD–G.
