# Supplementary material for: Multi–Omics Analysis of Key microRNA–mRNA Metabolic Regulatory Networks in Skeletal Muscle of Obese Rabbits
Source: Int J Mol Sci. 2021 Apr 19;22(8):4204. doi: 10.3390/ijms22084204 (PMC8072691; doi:10.3390/ijms22084204)
Supplement: Supplementary file 1 [file ijms-22-04204-s001.zip › Figure S1.docx]

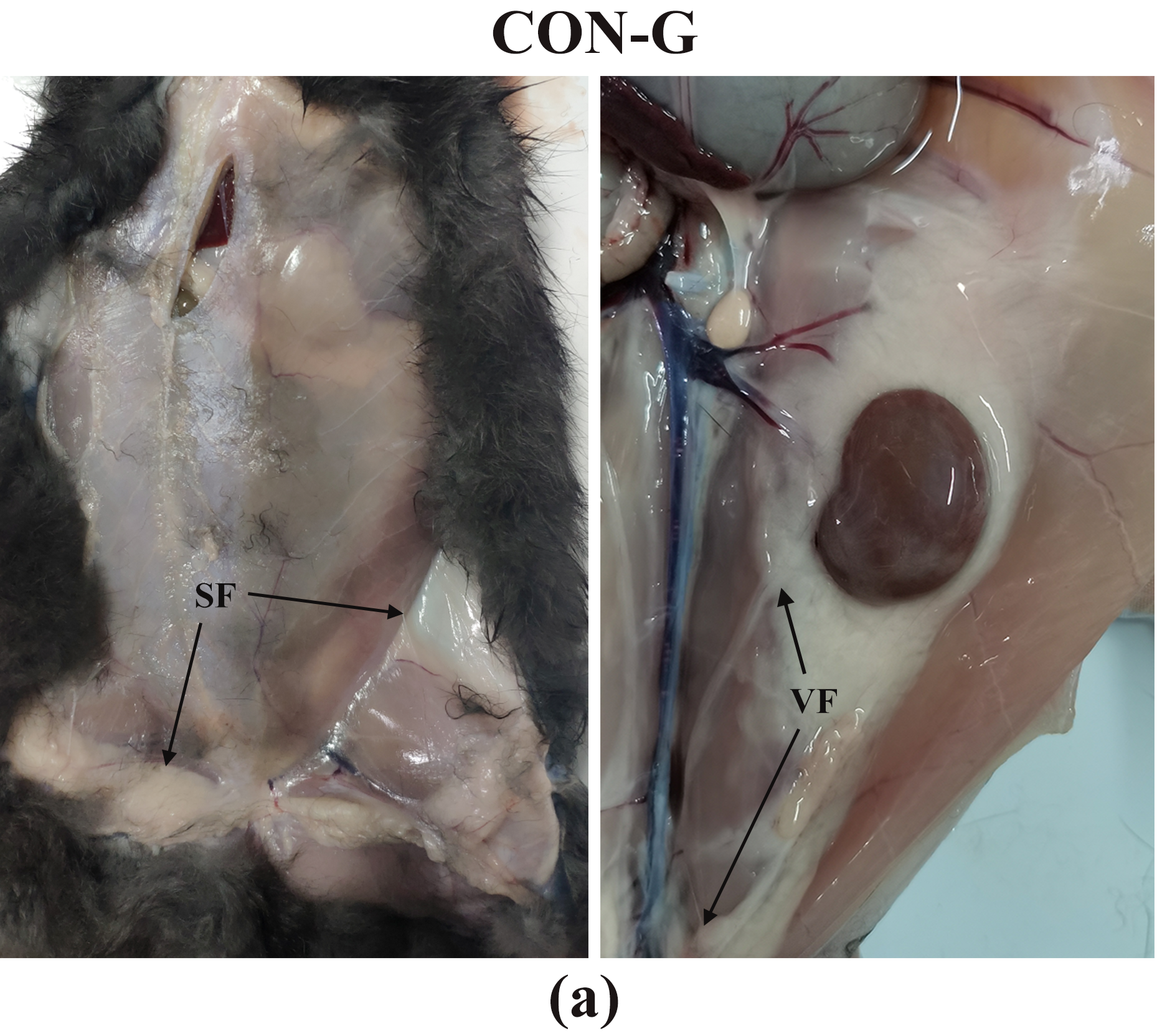

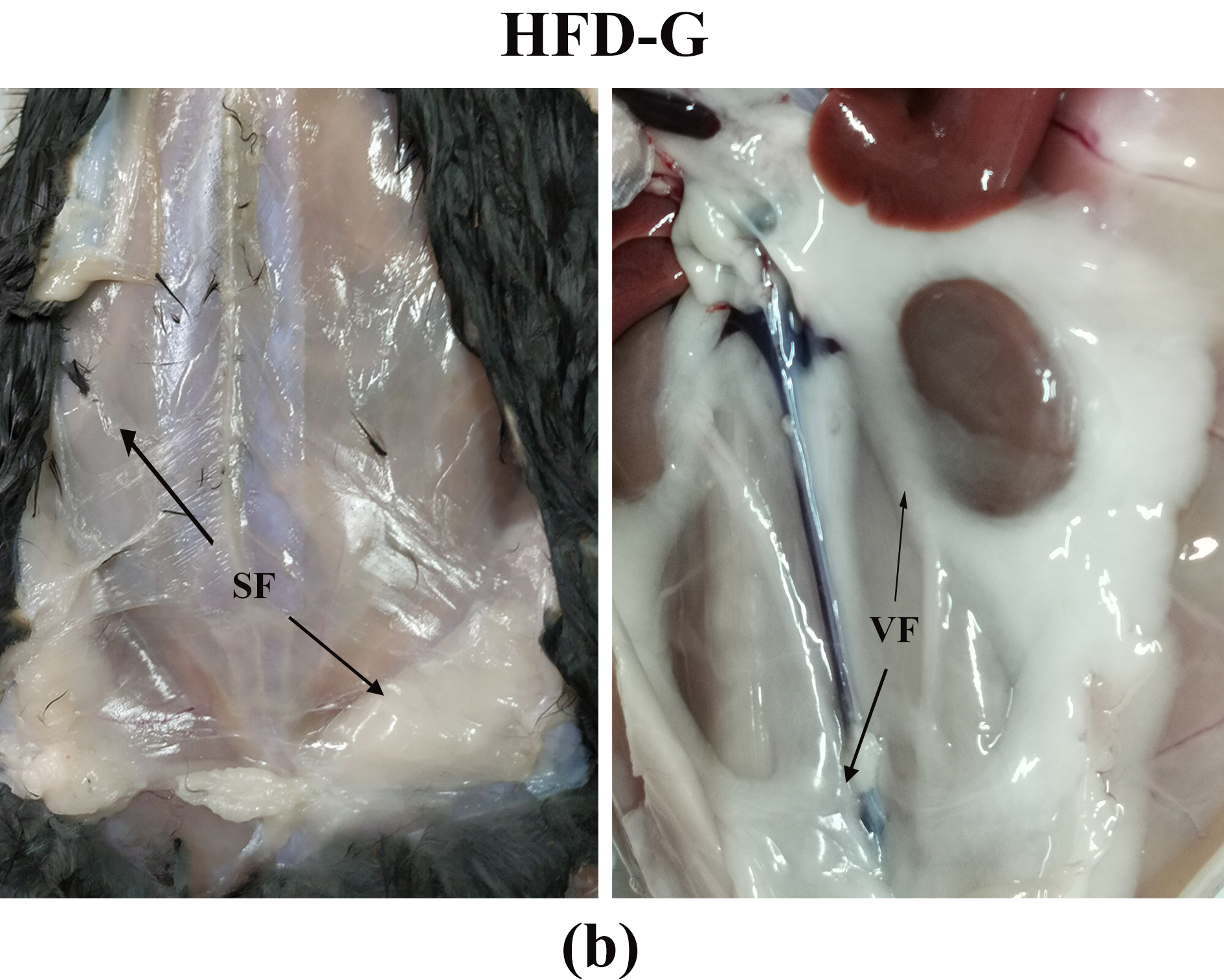


**Figure S1.** Anatomical differences of subcutaneous fat (SF) and visceral fat (VF) in rabbit between CON-G (**a**) and HFD-G (**b**).
